# Tumor tissue hnRNP M and HSP 90α as potential predictors of disease-specific mortality in patients with early-stage cutaneous head and neck melanoma: A proteomics-based study

**DOI:** 10.18632/oncotarget.27333

**Published:** 2019-11-19

**Authors:** Andro Košec, Ruđer Novak, Paško Konjevoda, Vladimir Trkulja, Vladimir Bedeković, Lovorka Grgurević

**Affiliations:** ^1^Department of Otorhinolaryngology and Head and Neck Surgery, University Hospital Centre Sestre Milosrdnice, Zagreb, Croatia; ^2^Department for Proteomics, Center for Translational and Clinical Research, School of Medicine, University of Zagreb, Zagreb, Croatia; ^3^Division of Molecular Medicine, Laboratory for Epigenomics, Rudjer Boskovic Institute, Zagreb, Croatia; ^4^Department of Pharmacology, School of Medicine, University of Zagreb, Zagreb, Croatia; ^5^Department of Anatomy “Drago Perović”, School of Medicine, University of Zagreb, Zagreb, Croatia; ^*^These authors contributed equally to this work

**Keywords:** melanoma, early stage, proteomics, tissue, biomarkers

## Abstract

Background: Breslow tumor thickness and mitotic rate are standardly used for risk stratification of patients with malignant melanoma. However, their prognostic value is relatively limited and a need for improved prognostication has been advocated. We aimed to screen the tumor tissue proteome in a search for potentially useful prognostic factors in early-stage cutaneous head and neck melanoma.

Methodology and Findings: Proteomic profiles of archival formalin-fixed tissue samples of 31 patients (age 23–90 years) with early-stage head and neck cutaneous malignant melanoma (American Joint Committee on Cancer, AJCC, stage I/II) were determined and expression intensities were compared to those of melanocytic nevi, yielding ratios used in data analysis. Medical charts were retrospectively reviewed to determine time elapsed since diagnosis to disease-specific death or censoring. In a multivariate recursive partitioning analysis (as *per* AJCC guidelines), higher expression levels of heterogeneous nuclear ribonucleoprotein M (hnRNP M) [*n =* 18, HR = 1.94 vs. the entire cohort; HR = 5.95 (95%CI 2.43–14.5) for “high” vs. “low” (*n =* 13)] and of heat shock protein 90 alpha (HSP 90α) [*n =* 17, HR = 2.09 vs. the entire cohort; HR = 4.59 (95%CI 1.87–11.2) for “high” vs. “low” (*n =* 14)] were each independently strongly associated with higher mortality (accounting for clinical and standard pathohistological features). Higher Breslow thickness and mitotic rate were associated with higher mortality only when proteomic data were disregarded.

Conclusions and Significance: Data suggest that tumor tissue expression of hnRNP M and/or of HSP 90α deserve further investigation and clinical validation as potential novel risk stratification aids in patients with stage I-II cutaneous head and neck malignant melanoma.

## INTRODUCTION

Cutaneous malignant melanoma is one of the most aggressive malignancies that accounts for 4% of skin tumors and is responsible for 80% of deaths related to skin tumors worldwide [1]. Epidemiological data indicate a markedly increasing (rate estimated at 2.6–9.5%) annual incidence of early stage melanoma [1]. Due to direct insolation, almost 20% of malignant melanomas are cutaneous head and neck melanomas (CHNM). Affected patients have poorer survival compared to patients with melanomas occurring in other parts of the body due to abundant lymphatic drainage pathways in the neck and early development of metastatic disease [2, 3]. According to the American Joint Committee on Cancer (AJCC), CHNM stages I and II are non-metastatic, and are stratified according to Breslow tumor thickness: stage I = tumor thickness below 2 mm; stage II = tumor thickness ≥2.01 mm [4]. Early risk stratification and adequate surgical treatment considerably improve prognosis in these patients. The AJCC staging system suggests several prognostic and predictive biomarkers to be used clinically in melanoma patients. Lactate dehydrogenase (LDH) is the only potential circulating biomarker whose elevated levels are associated with poor survival in stage IV malignant melanoma [5]. Other circulating proteins such as S100β, C-reactive protein (CRP) and melanoma inhibiting activity protein (MIA) have also shown diagnostic and prognostic potential in melanoma patients, but with limitations in routine clinical use due to significant intra- and inter-patient variation, limitations in specificity and sensitivity of current technology and lack of consistency in blood sampling, storage and processing [6]. Combined use of tumor type M2 pyruvate kinase (PKM2) and S100β improves the estimation of disease prognosis in metastatic melanoma patients, compared to the use of S100β alone [7].

Proteomic methods enable simultaneous large-scale identification and quantification of proteins from complex tissue samples. In melanoma research, plasma and serum proteomics emerged in the late 2000s, when platelet basic protein precursor was identified as predictive of survival in melanoma patients [8]. Recently, serum amyloid A protein was suggested as a prognostic factor in the early stages of melanoma [9]. Proteomic analyses of 69 lymph nodes pathohistologically positive for metastatic melanoma and 17 disease-negative lymph nodes showed that proteomic profiling could differentiate between metastatic and healthy tissues and accurately “recognize” clinical stage of the disease [10]. In 2010 and 2011 respectively, Rezaul and Byrum both published proof-of-principle techniques related to protein extraction from formalin-fixed paraffin embedded (FFPE) tissues [11, 12]. Introduction of proteomic methods is expected to improve CHNM staging and prognostics based on patient serum proteomic profiles [13, 14]. Identification of molecules involved in disease progression is the prerequisite for development of adequate prognostic tools and treatments for patients with a high-risk of metastatic melanoma [15]. Timely differentiation of high-risk patients would have a positive impact on the development of individualized patient follow-up strategies and help in early detection of metastatic disease [16]. Although many candidate molecules have been investigated, currently no biomarker can predict disease outcome in patients with early stage cutaneous melanoma.

To the best of our knowledge, proteomic tumor tissue expression profiles have not yet been related to patient survival in early stages of CHNM. In this study, we screened the tumor tissue proteome in archival FFPE samples of 31 patients with stage I and stage II CHNM in a search for potentially useful prognostic factors.

## RESULTS

**Figure 1:**
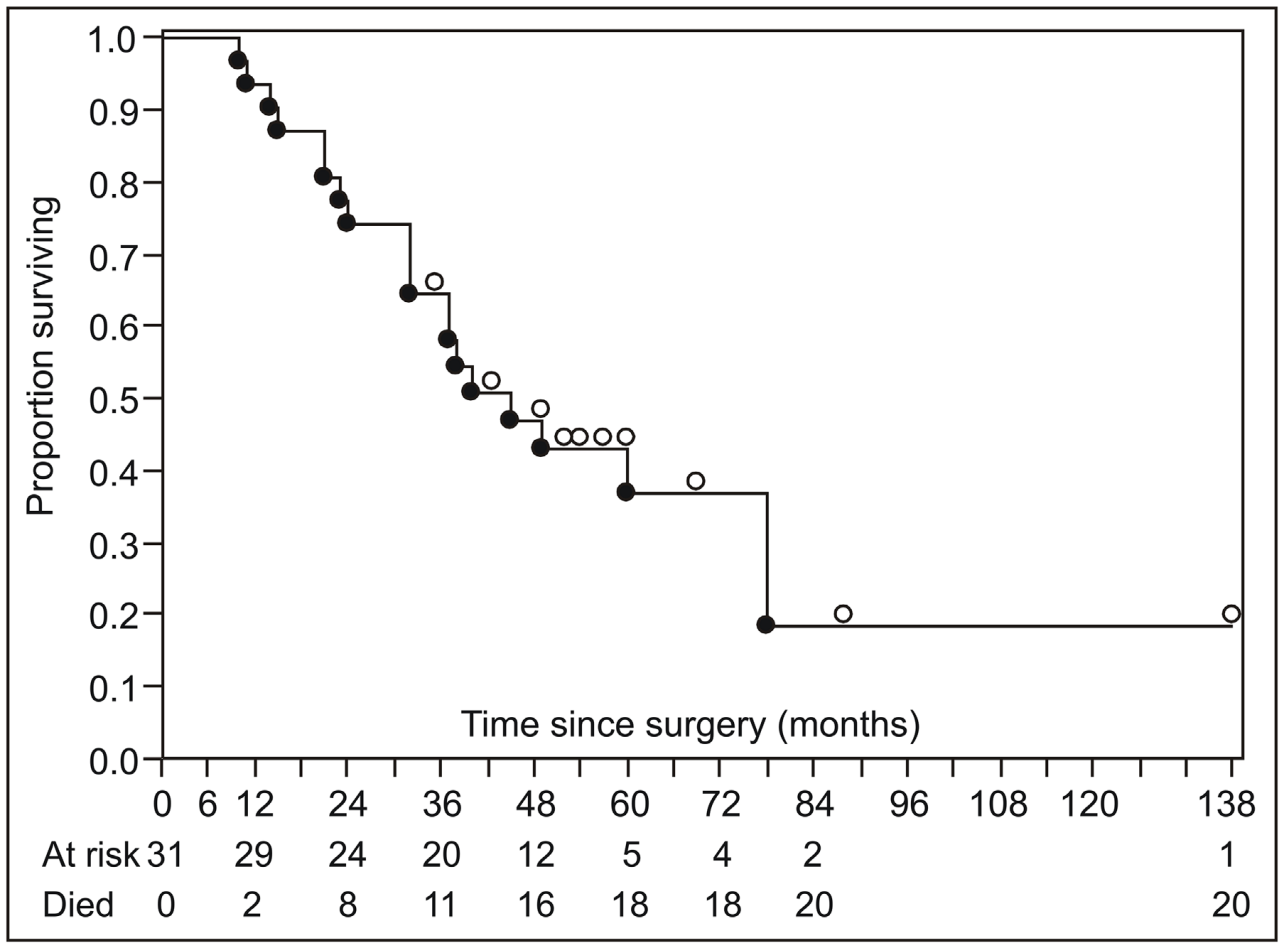
Summary of disease-specific survival (Kaplan–Meier product limit survival curve). Open circles above the survival curve indicate censored subjects; closed circles below the line indicate disease-specific deaths.

The cohort comprised 31 patients (18 men; age range 23–90 years), 15 of whom suffered from nodular melanoma, 12 from superficial spreading melanoma and 4 suffered from *lentigo maligna* melanoma. A total of 20 (64.5%) patients died during the observed period with first death occurring 10 months after the surgery. Patient characteristics and survival are summarized in Table 1 and Figure 1, respectively (see Supplementary Table 1 for individual patient data).

**Table 1:** Patient and tumor characteristics (overall, by gender and histological type of cutaneous head and neck melanoma)

		By gender	
	All patients	Men	Women
N	31	18 (58.1%)	13 (41.9%)
Age (years)	73 (23–90)	73 (44–90)	73 (23–84)
Nodular melanoma	15 (48.4%)	9 (50.0%)	6 (46.2%)
Superficial spreading melanoma	12 (38.7%)	8 (44.4%)	4 (30.8%)
*Lentigo maligna* melanoma	4 (12.9%)	1 (5.6%)	3 (23.0%)
Breslow thickness (mm, average)	3.37	3.62	3.01
Breslow stage 1/2/3/4/5	5/2/12/9/3	1/1/8/7/1	4/1/4/2/2
Clark stage 1/2/3/4/5	0/4/9/16 /2	0/1/5/10/2	0/3/4/6/0
Stage I/II	10/21	5/13	5/8
Mitotic rate (average)	5.5	5.3	5.9
T category			
1a or 1b	6	1	5
2a or 2b	4	4	0
3a or 3b	13	8	5
4a or 4b	8	5	3

Data are presented as count (%) or median (range).

**Figure 2:**
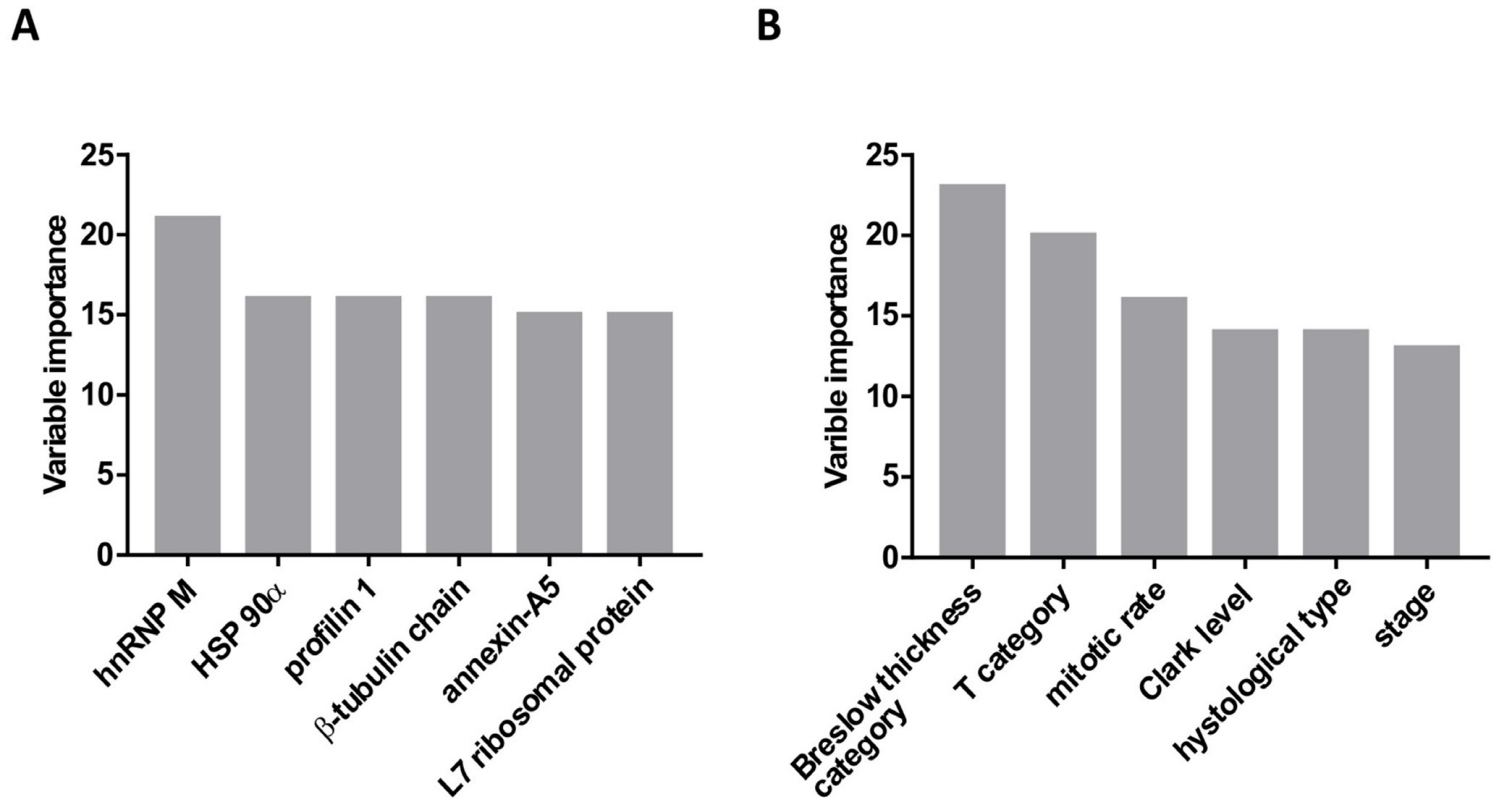
Variable importance (based on the strength of association with the mortality data) determined using the recursive partitioning method. (**A**) Analysis included all potential predictors (proteomic and clinico-pahtological). Only protein expression data were identified as “important variables”. (**B**) Analysis included only clinical and pathohistological data, without the protein expression data.

**Figure 3:**
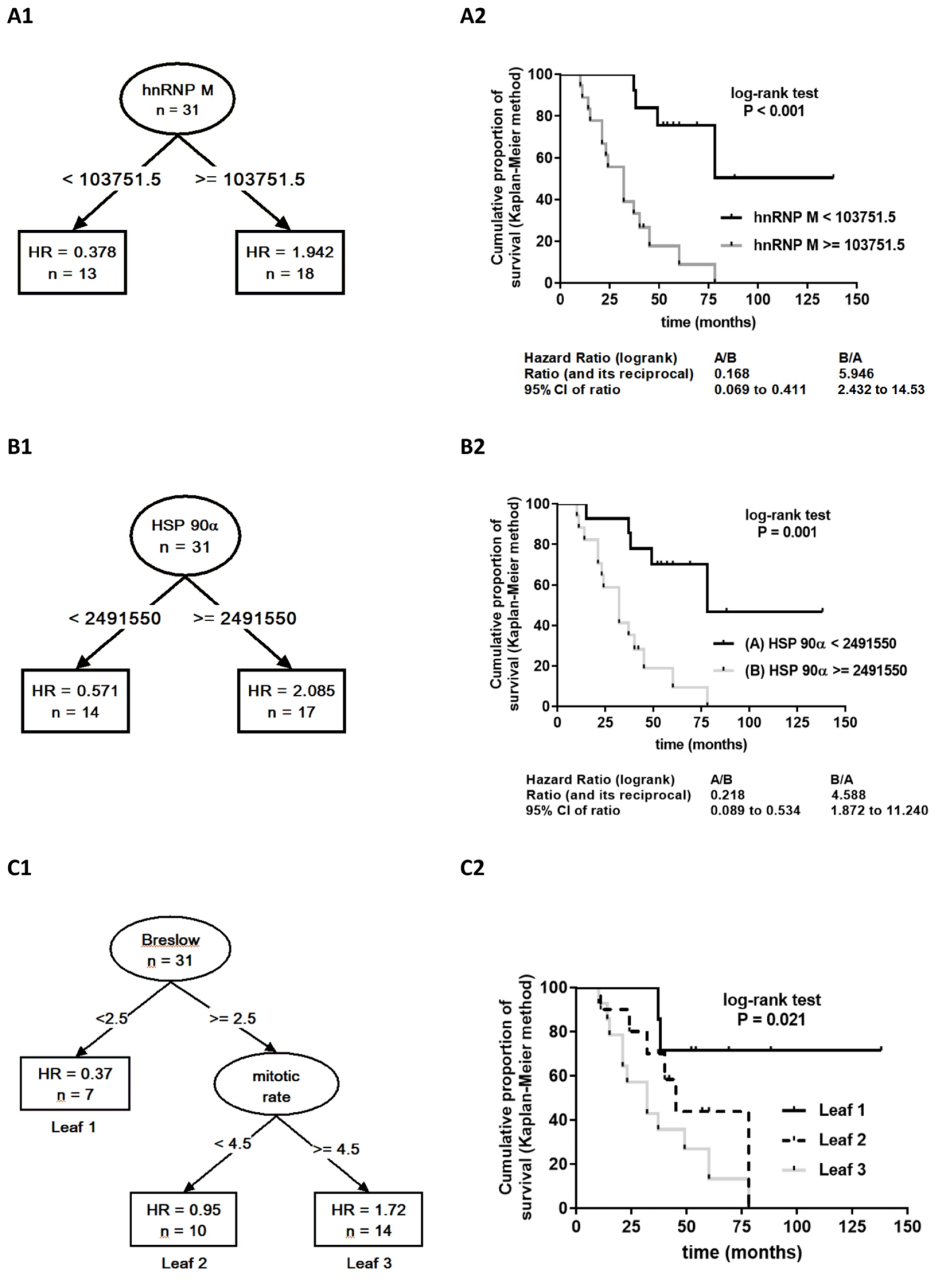
Results of the recursive partitioning survival data analysis presented as survival trees (**A1**, **B**, **C1**) with hazard ratios (HR) in the terminal nodes (leaves) [HR for the patients in the respective subset (leaf), relative to the entire cohort (starting node, *n* = 31, HR = 1.0). If leaf HR <1.0 – mortality lower than in the entire cohort; if >1.0 – mortality higher than in the entire cohort], and as Kaplan–Meier product limit survival curves for patients within the respective leaves generated by splitting of the starting node (**A2**, **B2**, **C2**) with log-rank HR and the associated *p*-value. A1 and A2. The analysis included all proteomic expression data and clinico-pathological data as potential predictors. The entire cohort (initial node) was split only once, based on the expression level of heterogeneous nuclear ribonucleoprotein M (hnRNP M): to patients with iBAQ below or above the identified cut-off iBAQ value. (**B1** and **B2**) The analysis was repeated whithout hnRNP M expression data: the entire cohort was split only once, based on the expression level of heat shock protein 90α (HSP 90α) to a subset with iBAQ below (HR<1.0) or above (HR>1.0) the identified cut-off value. (**C1** and **C2**) The analysis considered only clinico-pathohistological variables as potential predictors. The entire cohort was split based on Breslow thickness (cut-off 2.5 mm) and, if thickness ≥2.5 mm, also based on mitotic rate (cut-off 4.5) into three leaves with lower (leaf 1), comparable (leaf 2) or higher (leaf 3) mortality than for the entire cohort.

Across all samples, 1140 proteins were identified. Less reliable single peptide identifications were excluded as well as proteins that were not expressed across all samples. Finally, 47 proteins identified by at least two peptides were considered for statistical analysis (Supplementary Table 2, Supplementary Table 3). Considering all proteomic, pathohistological and clinical data, recursive partitioning procedure identified exclusively proteomic variables (6 out of 47 considered proteins) as relevant for prediction of survival, with expression level of heterogeneous nuclear ribonucleoprotein M (hnRNP M) as the most relevant one, followed by heat shock protein 90α expression level (HSP 90α) (Figure 2A). Other identified important variables were expression levels of profilin-1, β-tubulin chain, annexin-A5, and L7 ribosomal protein (Figure 2A) – their association with survival appeared stronger than association of any of the clinical/pathological variables (Breslow tumor thickness category, mitotic rate, T-category, histological tumor type or disease state). When proteomic data were disregarded, Breslow thickness category appeared the most relevant predictor of survival (Figure 2B). Considering all potential predictors, the entire cohort was split only once, into two subsets based on hnRNP M expression levels: the procedure identified a cut-off iBAQ value that split the cohort in subsets below (*n* = 13) and above the cut-off (*n* = 18) (Figure 3A1). In respect to the entire cohort (reference, with hazard ratio, HR = 1.0), the subset with lower hnRNP M expression had lower mortality risk (HR = 0.378), and the subset with higher hnRNP M expression had higher risk (HR = 1.94) (Figure 3A1). Disease-specific mortality was considerably higher in patients with hnRNP M expression above the cut-off (“high”) than in patients with expression below the cut-off (“low”) – HR = 5.95, log-rank *P* < 0.001 (Figure 3A2). When hnRNP M was omitted from the analysis, the cohort was again split only once, into two subsets based on the HSP 90α expression level (Figure 3B1): again, the subset below the cut-off iBAQ value (“low”, *n* = 14) had lower mortality vs. the entire cohort (HR = 0.571), while the subset above the cut-off value (“high”, *n* = 17) had higher mortality vs. the entire cohort (HR = 2.085) (Figure 3B1). Disease-specific mortality was also strongly associated with higher expression level of HSP 90α (HR = 4.588, log-rank *P* = 0.001) (Figure 3B2). When all proteomic data were disregarded, the cohort was split based on the Breslow thickness category into a subset with thickness <2.5 (leaf 1, *n* = 7), and a subset with thickness ≥2.5 which was further split into a subset with mitotic rate <4.5 (leaf 2, *n* = 10) and a subset with mitotic rate ≥4.5 (leaf 3, *n* = 14). Compared to the entire cohort, mortality was lower (HR = 0.37), comparable (HR = 0.95) or higher (HR = 1.72) in the three respective subsets (leaves) (Figure 3C1). It was higher in patients presented by leaf 3 patients vs. patients presented by leaf 1 (Figure 3C2). Table 2 summarizes patient characteristics across the subsets formed in these procedures.

**Table 2:** Patient characteristics across subsets based on heterogeneous nuclear ribonucleoprotein M (hnRNP M) expression level, heat shock protein 90α (HSP 90α) expression level and subsets (leafs) based on Breslow thickness and mitotic rate identified by the recursive partitioning analysis of disease-specific mortality

	hnRNP M expression cut-off iBAQ 103751.5		HSP 90α expression cut-off iBAQ 2491550		Breslow thickness (cut-off 2.5) and mitotic rate (cut-off 4.5)		
	“Low”	“High”	“Low”	“High”	Leaf 1	Leaf 2	Leaf 3
N	13	18	14	17	7	10	14
Men	6	12	7	11	2	7	9
Women	7	6	7	6	5	3	5
Nodular melanoma	2	13	3	13	0	5	10
Superficial spreading melanoma	7	5	7	4	3	5	4
*Lentigo maligna* melanoma	4	0	4	0	4	0	0
Breslow thickness (mm)	1.82	4.49	1.81	4.65	0.75	3.65	4.48
Breslow stage 1/2/3/4/5	5/2/4/2/0	0/0/8/7/3	5/2/5/2/0	0/0/7/7/3	5/2/0/0/0	0/0/6/2/2	0/0/4/2/8
Stage I/II	8/5	2/16	10/4	0/17	7/0	0/10	0/14
Mitotic rate	2.9	7.5	3.3	7.4	2	3.2	9
Median iBAQ hnRNP M	21468	537915	24400.5	559600	17142	425325	395375
Median iBAQ HSP 90α	407380	4664450	458345	4693600	176980	2794250	3229550
T category							
1a or 1b	6	0	6	0	6	0	1
2a or 2b	2	2	4	0	1	1	2
3a or 3b	3	10	2	11	0	7	6
4a or 4b	2	6	2	6	0	2	6

Based on Breslow thickness and mitotic rate, patients were split into leaf 1 (Breslow thickness <2.5), leaf 2 (Breslow thickness ≥2.5 and mitotic rate <4.5) and leaf 3 (Breslow thickness ≥2.5 and mitotic rate ≥4.5) (Figure 3). Data are presented as count (%) or median (range) unless specified otherwise.

## DISCUSSION

Cutaneous melanoma, particularly in the head and neck region (CHNM), is one of the most aggressive malignancies with continuously increasing incidence worldwide [1]. Successful treatment and favorable prognosis rely upon accurate and early tumor staging and risk stratification [2]. Breslow melanoma thickness is currently the most reliable prognostic factor for localized CHNM before metastases have occurred. Still, Breslow thickness cannot reliably identify patients with a high risk of metastatic disease due to cut-off clustering. Accordingly, it is not surprising to see that many patients with low Breslow thickness develop metastatic disease [17, 18]. Occult metastatic regional disease develops in 16% of stage I and II patients. Adequate surgical or adjuvant treatment is then needed, which rarely happens based on currently used clinical criteria [19]. Serum levels of several proteins, such as S100β, CRP, melanoma inhibitory activity (MIA) protein and LDH have been suggested as early biomarker candidates, but they do not seem to improve patient sub-classification into specific prognostic groups [20–22]. Melanoma tissue proteins appear to hold some promise in this respect. High expression of syntaxin 7 in melanoma tissues was shown to be inversely proportional to tumor growth and aggressiveness [23], while a proteomics-based study identified several molecules in metastatic tissue, potentially promising regarding predictions of metastatic disease [24]. So far, however, no study addressed early-stage CHNM and prediction of disease-specific survival. In this respect, archival tissues are a valuable source of information, particularly in the light of technological developments enabling proteomic analysis of FFPE samples [24, 25]. In the present pilot study, we screened the proteomic profile of FFPE tumor tissue samples from 31 adult patients with early-stage CHNM for whom disease-specific survival was determined retrospectively. We detected signals for more than a thousand different proteins, 47 of which could be reliably identified across all individual samples, and were considered combined with standard clinico-pathological indices as potential predictors of mortality. In this highly dimensional dataset, recursive partitioning algorithm depicted only proteomic variables as important regarding association with mortality: a set of six highly expressed proteins was identified as “important variables”. In particular, expression levels of hnRNP M and of HSP 90α were revealed as parameters with the strongest association with the outcome: “high” expression (i.e., above the cut-off values of arbitrary expression intensity units detected by the algorithm) was associated with several-fold higher mortality than the “low” expression. At the same time, classical clinico-pathological indices, primarily Breslow thickness and mitotic rate (combined thickness ≥2.5 and mitotic rate ≥4.5 – higher mortality) became relevant predictors only when proteomic data were disregarded. Moreover, the strength of association between hnRNP M and HSP 90α expression signals and the outcome, appeared “stronger” than that of Breslow thickness/mitotic rate in the sense of numerically higher hazard ratios. These observations strongly suggest hnRNP M and HSP 90α tissue expression levels as potentially helpful aids in risk stratification of early-stage CHNM. Biologically, this also seems plausible. HnRNP M is a ubiquitously expressed RNA-binding protein involved in stabilization, splicing, transcription and translation of RNA (https://www.proteinatlas.org/ENSG00000099783-HNRNPM/pathology). Through mRNA processing, as a part of the spliceosome machinery, it regulates the expression of many proteins and its disruption can promote proliferation, invasion, and metastasis of tumor cells. It was proposed as a predictive factor for colorectal, ovarian and breast cancer [25, 26]. This protein was shown to dramatically increase breast cancer xenograft tumor growth [27]. HSP 90α is a chaperone essential for stabilization and/or activation of hundreds of cellular proteins. Its expression increases in almost all cancer types: the cancer cell is in a way dependent to HSP 90 proteins that commonly stabilize mutated tumor-related proteins and stimulate malignant transformation [27]. Although increased serum HSP 90α expression levels have been identified in metastatic melanoma patients, its prognostic significance in relation to early-state disease survival has not been demonstrated. Present data suggest tumor tissue HSP 90α (high) expression as a potential marker of poor prognosis [28].

There are two major limitations of the present study. A small single-center sample clearly cannot fully represent variability (and contributing factors, their “overlaps” and interactions) of survival in early-stage CHNM patients, or of expression (qualitative and quantitative) of particular tumor tissue proteins. Consequently, small samples might recognize only very prominent effects (regarding the clinical outcome), which could occur by chance, while some weaker, but potentially clinically relevant effects might be omitted. The limitations of the employed proteomics approach (i.e., those beyond potential technological limitations of mass spectrometry, as its performance is given “as is” to any researcher) lie with the fact that it was focused solely on identification of proteins with approximate quantification of intensity in arbitrary units that are of little practical relevance (at this point). In an attempt to reduce chance findings/signals, the false discovery rate was set to 1% at both the peptide and the protein level. Accordingly, we restricted our analysis to only those signals (proteins) that could be reliably identified in all patient samples as well as in control sample of pooled melanocytic nevi. By this virtue, we believe we have managed to avoid false signals, but again, this restrictive approach could have contributed to omission of some possibly relevant ones. Despite these limitations, we believe that the present data clearly support the need for further evaluation (e.g., immunohistological verification; accurate quantification; evaluation of their relationship to histological tumor types and clinico-pathological staging) of association of hnRNP M and/or of HSP 90α early-stage CHNM expression levels and the risk of metastases occurrence. The high expression of each of these two proteins is independently strongly associated with patient survival, and in a hierarchy of “important predictors” they appeared dominant to classical clinico-pathological factors.

However challenging integration of novel factors into the existing prognostic tools may be, this process is vital for improvement of clinical estimation of risk stratification in patients with melanoma.

## MATERIALS AND METHODS

### Study design

Ethics committee at the School of Medicine, University of Zagreb, approved the study. All included CHNM patients were treated at a single tertiary referral center between January 1st 2000 and December 31st 2012, and were postoperatively followed-up with regularly updated oncological status over a minimum of 60 months. Before surgery, all patients provided a signed informed consent and agreed to the use of tissue samples for research purposes. Their clinico-pathological stage was determined in line with the AJCC criteria [4] (see Supplementary Material, Supplementary Table 1 for individual data). For the purpose of the study, their medical charts were reviewed between January and March 2018 to retrospectively determine time elapsed since surgery to death due to disease progression (disease-specific mortality). Censored time was defined as time elapsed between surgery and chart review or death, not related to disease progression. Proteomic analysis was performed on 31 CHNM tissue samples patients, and also on a pooled tissue sample containing melanocytic nevi obtained from six patients without malignant melanoma (control sample) who were treated at the same center during the same time-period and under the same conditions as the CHNM patients.

Proteins were extracted from FFPE tissues using a commercial kit (FFPE-FASP kit, Expedeon) according to manufacturer’s instructions. Protein concentration was determined using RC DC Protein Assay Kit II (Biorad). Digested peptides were purified using a 30 kDa cut-off Spin Filter centrifuge column and concentrated using Stage Tips [29]. Peptides were separated on a C18 column by liquid chromatography (Easy-nLC, Proxeon Biosystems) and analyzed by mass spectrometry (LTQ Orbitrap Discovery, Thermo Scientific). Automated mass spectrometric measurement cycles consisted of full MS scanning and MS/MS scanning of up to twenty most intense ions. Full MS scans ranging from m/z 300 to 2,000, were obtained in the Orbitrap analyzer at a resolution of 100,000, with internal calibration of the instrument using the lock mass setting. MaxQuant software version 1.5.1.2. (Max Planck Institute of Biochemistry) was used to process the raw data and quantify the detected proteins using intensity-based absolute quantification (iBAQ) algorithm [30]. Trypsin was selected for *in silico* digestion, carbamidomethylation, N-terminal acetylation and methionine oxidation were used as variable peptide modifications. No fixed modifications were specified. False discovery rate at the peptide spectrum level and at the protein detection level was set at 1%. Minimum peptide length for protein identification was seven amino acids. The main search peptide mass tolerance was set to 4.5 ppm. Common laboratory contaminants were excluded from the analysis. Proteins were quantified using intensity-based absolute quantification (iBAQ), a continuous intensity value of protein expression in individual samples (i.e. the ratio of the sum of the experimentally determined intensities of all peptides and the intensity of the individual detected peptide). Experimental data was compared with the set of human proteins available in the UniProt database (http://www.uniprot.org). Samples were analyzed in technical triplicates and proteins identified with at least two peptides in all samples were considered relevant for statistical analysis. The mass spectrometry proteomics data have been deposited to the ProteomeXchange Consortium (http://proteomecentral.proteomexchange.org) via the PRIDE partner repository with the dataset identifier PXD015137 [31].

### Statistical rationale

Only proteins identified in all patient samples and in the control sample were considered for data analysis. iBAQ values for each protein and each individual patient were compared to the respective iBAQ value of the control sample to yield proportionality ratios used in data analysis. Considering high dimensionality of data (a number of proteomic and clinico-pathological variables as potential predictors), multivariate recursive partitioning (RP) algorithm was implemented to analyze survival data [rpart module within the programming language R (R Foundation for Statistical Computing, Vienna, Austria URL https://www.R-project.org/) and SPSS (Version 22.0 released in 2013 IBM SPSS Statistics for Windows, Armonk, NY: IBM Corp.)] [32–34]. RP-based programs clarify complex and non-linear interactions, and enable robust conclusions in high-dimensional data sets. They are increasingly used in oncology for extracting risk factors, developing prognostic indexes, and optimizing diagnostic procedures and treatments [35, 36]. The result of recursive partitioning is presented as a survival tree, which begins with a starting node. All patients are included in the starting node, and their hazard ratio (HR) is 1. Using different cut-off values the starting patient group is partitioned in subgroups in one or more decision steps. The final nodes (leaves) correspond to subgroups with maximal difference in identified HRs. It is important to note that their HR is expressed in comparison to the starting node. The comparison of final nodes (leaves) was done using Kaplan–Meier survival curves and log-rank test. All statistical tests were two-sided. The *p* values ≤ 0.05 were considered statistically significant.

## SUPPLEMENTARY MATERIALS



## Abbreviations

CHNM: Cutaneous head and neck melanomas
AJCC: American Joint Committee on Cancer
LDH: Lactate dehydrogenase
CRP: C-reactive protein
PKM2: Tumor type M2 pyruvate kinase
FFPE: Formalin-fixed paraffin embedded
iBAQ: Intensity-based absolute quantification
HR: Hazard ratio
hnRNP M: Heterogeneous nuclear ribonucleoprotein M
HSP 90α: Heat shock protein 90 alpha
MIA: Melanoma inhibitory activity
