# Supplementary material for: Tumor tissue hnRNP M and HSP 90α as potential predictors of disease-specific mortality in patients with early-stage cutaneous head and neck melanoma: A proteomics-based study
Source: Oncotarget. 2019 Nov 19;10(62):6713–22. doi: 10.18632/oncotarget.27333 (PMC6877100; doi:10.18632/oncotarget.27333)
Supplement: Supplementary file 1 [file oncotarget-10-6713-s001.pdf]

Tumor tissue hnRNP M and HSP 90α as potential predictors of disease-specific mortality in patients with early-stage cutaneous head and neck melanoma: A proteomics-based study

SUPPLEMENTARY MATERIALS

Supplementary Table 1: Individual patient data

Patient No	Breslow thickness (mm)	Mitotic rate	T category	Stage	Breslow stage	Clark stage	Tumor Invading Lymphocytes	Follow-up (months)	Census
1	0.6	2	1a	I	1	2	2	69	1
2	0.7	0	1a	I	1	2	2	54	1
3	1	4	1a	I	2	3	2	138	1
4	0.49	1	1a	I	1	2	1	37	0
5	0.65	2	1a	I	1	4	3	88	1
6	0.3	2	1b	I	1	2	3	52	1
7	2	4	2a	I	3	4	1	24	0
8	1.74	6	2a	I	3	4	2	49	1
9	1.48	3	2a	I	2	3	2	38	0
10	1.73	6	2a	I	3	3	3	15	0
11	2.39	3	3a	II	4	3	2	32	0
12	2.25	4	3a	II	3	4	2	42	1
13	2.2	6	3a	II	3	4	2	23	0
14	2.25	4	3a	II	3	3	2	40	0
15	2.5	4	3a	II	3	3	2	78	0
16	2	1	3a	II	3	3	1	57	1
17	2.1	3	3a	II	3	4	2	60	1
18	2.2	10	3b	II	3	3	3	32	0
19	3.3	15	3b	II	5	3	2	21	0
20	3	2	3b	II	4	4	3	45	0
21	2.39	6	3b	II	3	4	2	32	0
22	3.19	8	3b	II	5	4	1	60	0
23	2.39	16	3b	II	3	4	3	37	0
24	7	13	4a	II	4	4	2	21	0
25	7	5	4a	II	4	5	1	10	0
26	6	3	4a	II	4	4	3	37	1
27	4.1	6	4a	II	4	4	1	49	0
28	6	10	4a	II	4	5	1	14	0
29	5.44	12	4b	II	4	4	3	78	0
30	12	4	4b	II	5	4	1	11	0
31	14	7	4b	II	4	4	2	37	1

Census: 1 - alive at the end of follow-up, 0 – disease-specific death.

Supplementary Table 2: Proteins represented by at least two peptides, expressed in all melanoma samples with iBAQ values (arbitrary units) obtained by proteomic analysis

Protein name	Uniprot accession no.	Number of peptides	Average iBAQ	Median iBAQ	Standard deviation
14-3-3 protein zeta/delta	P63104	10	1.43E+13	1.03E+07	1.36E+13
40S ribosomal protein S16	M0R210	6	7.13E+12	4.65E+06	6.31E+12
60S ribosomal protein L7	***P18124***	7	2.87E+12	1.03E+06	3.47E+12
Actin, alpha cardiac muscle 1	P68032	19	7.89E+12	2.87E+06	2.14E+13
Actin, cytoplazmatic 2	P63261	20	1.71E+14	1.36E+08	1.35E+14
Alpha-enolase	P06733	16	7.86E+12	4.54E+06	8.25E+12
Annexin A1	P04083	16	3.46E+12	9.43E+05	4.84E+12
Annexin A5	***P08758***	15	2.80E+13	1.21E+07	3.17E+13
ATP synthase subunit alpha, mitochondrial	P25705	24	6.90E+12	4.06E+06	1.26E+13
ATP synthase subunit beta, mitochondrial	P06576	20	6.11E+12	3.69E+06	1.02E+13
Calmodulin	P62158	4	8.72E+12	6.59E+06	1.10E+13
Cathepsin D	A0A1B0GW44	11	4.56E+12	1.25E+06	7.24E+12
Cathepsin G	P08311	6	1.16E+13	6.48E+06	1.33E+13
Cofilin-1	P23528	5	6.57E+12	3.34E+06	8.79E+12
Collagen alpha-1(VI) chain	A0A087X0S5	18	2.62E+12	1.29E+06	3.43E+12
Collagen alpha-3(VI) chain	P12111-2	98	5.05E+12	2.86E+06	6.50E+12
Galectin-3	P17931	7	1.56E+13	1.08E+07	1.88E+13
Galectin-3-binding protein	Q08380	4	5.67E+11	2.44E+05	1.16E+12
Galectin-7	P47929	7	2.25E+13	1.26E+07	2.55E+13
Glyceraldehyde-3-phosphate dehydrogenase	P04406	12	1.89E+13	1.34E+07	1.80E+13
Heat shock 71 kDa protein	P11142	16	3.42E+12	2.74E+06	3.26E+12
Heat shock protein beta-1	P04792	13	4.54E+13	3.57E+07	4.73E+13
Heat shock protein HSP 90-alpha	***P07900***	12	3.32E+12	1.70E+06	4.17E+12
Hemoglobin subunit alpha	P69905	8	5.40E+14	2.10E+08	6.41E+14
Hemoglobin subunit beta	P68871	11	8.38E+14	3.15E+08	1.10E+15
Heterogeneous nuclear ribonucleoprotein M	***P52272-2***	9	4.27E+11	1.75E+05	5.47E+11
Histone H2A type 1-J	Q99878	7	1.05E+15	9.58E+08	5.56E+14
Histone H2B type 1-M	Q99879	9	4.13E+14	3.75E+08	2.75E+14
Histone H3	K7EK07	5	1.98E+14	1.13E+08	2.10E+14
Histone H4	P62805	13	6.92E+14	6.56E+08	3.43E+14
Ig kappa chain C region	P01834	3	1.05E+13	7.64E+06	1.06E+13
Keratin, type II cytoskeletal 1	P04264	34	1.17E+13	5.74E+06	1.43E+13
Keratin, type II cytoskeletal 5	P13647	39	2.14E+13	2.04E+07	1.83E+13
Peptidyl-prolyl cis-trans isomerase A	P62937	12	2.28E+13	1.27E+07	2.53E+13

Peroxiredoxin-2	P32119	13	6.53E+12	5.35E+06	5.78E+12
Phosphoglycerate kinase 1	P00558	13	4.38E+12	2.67E+06	4.67E+12
Profilin-1	***P07737***	7	1.27E+13	8.13E+06	1.44E+13
Protein S100-A7	P31151	7	4.82E+12	2.99E+06	4.79E+12
Protein S100-A8	P05109	9	1.09E+13	8.50E+06	1.04E+13
Protein S100-A9	P06702	9	1.99E+13	9.22E+06	3.03E+13
Putative elongation factor 1-alfa-like 3	Q5VTE0	8	2.48E+13	1.60E+07	3.27E+13
Pyruvate kinase isoform 2	P14618	19	4.52E+13	2.28E+07	5.96E+13
Serum albumin	P02768	30	6.62E+13	4.60E+07	6.04E+13
Tubulin alpha-1B chain	P68363	15	2.04E+13	1.04E+07	1.93E+13
β -Tubulin chain	***Q5JP53***	18	1.85E+13	1.14E+07	2.03E+13
Ubiquitin-60S ribosomal protein	J3QS39	3	3.30E+13	2.22E+07	2.70E+13
Vimentin	P08670	51	9.32E+13	6.71E+07	7.35E+13

Proteins depicted in bold-italic font are those found by the recursive partitioning procedure as being of importance in definition of nodes.

Supplementary Table 3: Proteins represented by at least two peptides, expressed in the control sample, with iBAQ values (arbitrary units) obtained by proteomic analysis

Protein name	Uniprot accession no.	Number of peptides	iBAQ
14-3-3 protein zeta/delta	P63104	10	17230000
40S ribosomal protein S16	M0R210	6	7393300
60S ribosomal protein L7	***P18124***	7	742970
Actin, alpha cardiac muscle 1	P68032	19	1900300
Actin, cytoplazmatic 2	P63261	20	202050000
Alpha-enolase	P06733	16	8598800
Annexin A1	P04083	16	2561500
Annexin A5	***P08758***	15	19850000
ATP synthase subunit alpha, mitochondrial	P25705	24	12322000
ATP synthase subunit beta, mitochondrial	P06576	20	6182300
Calmodulin	P62158	4	13345000
Cathepsin D	A0A1B0GW44	11	13962
Cathepsin G	P08311	6	8056300
Cofilin-1	P23528	5	3682000
Collagen alpha-1(VI) chain	A0A087X0S5	18	9910100
Collagen alpha-3(VI) chain	P12111-2	98	10558000
Galectin-3	P17931	7	5174000
Galectin-3-binding protein	Q08380	4	18732

Galectin-7	P47929	7	122710000
Glyceraldehyde-3-phosphate dehydrogenase	P04406	12	11711000
Heat shock 71 kDa protein	P11142	16	3694700
Heat shock protein beta-1	P04792	13	90459000
Heat shock protein HSP 90-alpha	***P07900***	12	5715800
Hemoglobin subunit alpha	P69905	8	697170000
Hemoglobin subunit beta	P68871	11	918610000
Heterogeneous nuclear ribonucleoprotein M	***P52272-2***	9	93482
Histone H2A type 1-J	Q99878	7	1886400000
Histone H2B type 1-M	Q99879	9	568740000
Histone H3	K7EK07	5	236490000
Histone H4	P62805	13	1170000000
Ig kappa chain C region	P01834	3	20090000
Keratin, type II cytoskeletal 1	P04264	34	53502000
Keratin, type II cytoskeletal 5	P13647	39	149340000
Peptidyl-prolyl cis-trans isomerase A	P62937	12	16881000
Peroxiredoxin-2	P32119	13	8946000
Phosphoglycerate kinase 1	P00558	13	4412600
Profilin-1	***P07737***	7	17481000
Protein S100-A7	P31151	7	3764600
Protein S100-A8	P05109	9	716600
Protein S100-A9	P06702	9	1274300
Putative elongation factor 1-alfa-like 3	Q5VTE0	8	486720
Pyruvate kinase isoform 2	P14618	19	2956800
Serum albumin	P02768	30	107990000
Tubulin alpha-1B chain	P68363	15	28225000
β -Tubulin chain	***Q5JP53***	18	16301000
Ubiquitin-60S ribosomal protein	J3QS39	3	76320000
Vimentin	P08670	51	259730000

Proteins depicted in bold-italic font are those found by the recursive partitioning procedure as being of importance in definition of nodes.
